# Transcriptomic Analyses of the Adenoma-Carcinoma Sequence Identify Hallmarks Associated With the Onset of Colorectal Cancer

**DOI:** 10.3389/fonc.2021.704531

**Published:** 2021-08-11

**Authors:** Qin Hong, Bing Li, Xiumei Cai, Zhengtao Lv, Shilun Cai, Yunshi Zhong, Bo Wen

**Affiliations:** ^1^Key Laboratory of Metabolism and Molecular Medicine, Ministry of Education, Department of Biochemistry and Molecular Biology, School of Basic Medical Sciences, and Institutes of Biomedical Sciences, Fudan University, Shanghai, China; ^2^Endoscopy Center and Endoscopy Research Institute, Zhongshan Hospital, Fudan University, Shanghai, China; ^3^Key Laboratory of Metabolism and Molecular Medicine, Ministry of Education, Department of Biochemistry and Molecular Biology, School of Basic Medical Sciences, Fudan University, Shanghai, China

**Keywords:** RNA sequencing, *TPD52L1*, adenoma-carcinoma sequence, colorectal cancer, dynamic expression patterns

## Abstract

The concept of the adenoma-carcinoma sequence in colorectal cancer (CRC) is widely accepted. However, the relationship between the characteristics of the transcriptome and the adenoma-carcinoma sequence in CRC remains unclear. Here, the transcriptome profiles of 15 tissue samples from five CRC patients were generated by RNAseq. Six specific dynamic expression patterns of differentially expressed genes (DEGs) were generated by mFuzz. Weighted correlation network analysis showed that DEGs in cluster 4 were associated with carcinoma tissues, and those in cluster 6 were associated with non-normal tissues. Gene Ontology and Kyoto Encyclopedia of Genes and Genomes analyses identified metabolic dysregulation as a consistent finding throughout the transition process, whereas downregulation of the immune response occurred during normal to adenoma transition, and the upregulation of canonical pathways was associated with adenoma to carcinoma transition. Overall survival analysis of patients in cluster 6 identified *TPD52L1* as a marker of poor prognosis, and cell proliferation, colony formation, wound healing, and Transwell invasion assays showed that high expression levels of *TPD52L1* promoted malignant behaviors. In total, 70 proteins were identified as potential partners of hD53 by mass spectrometry. CRC formation was associated with three cancer hallmarks: dysregulation of metabolism, inactivation of the immune response, and activation of canonical cancer pathways. The *TPD52L1* gene was identified as a potential marker to track tumor formation in CRC and as an indicator of poor patient prognosis.

## Introduction

Colorectal cancer (CRC) is the fourth most frequently diagnosed cancer and the second most common cause of cancer-related death among men and women worldwide (1). Most cases of CRC are sporadic and arise from polyps originating within aberrant crypts. Approximately 10% of such polyps develop into early adenoma, followed by advanced adenoma, and finally CRC. Such progression is broadly categorized as either the traditional tubular adenoma route or serrated polyp route. The transition from tubular adenoma to adenocarcinoma occurs over a period of more than 10 to 15 years and is accompanied by sequential changes initially in the Wnt signaling pathway, followed by the RAS-RAF-MAPK, TGF-β, and PI3K-AKT pathways (2). The concept of polyp to cancer progression in the large bowel was first proposed in 1974 (3). An improved understanding of the diverse genetic and epigenetic changes that underlie the adenoma-carcinoma sequence was achieved after the development of a multi-hit genetic model of the carcinogenesis of CRC (4), as well as accumulating insight into chromosomal instability, microsatellite instability, and the CpG island methylator phenotype of CRC (5–7).

The emergence and development of omics technology has facilitated several systematic studies comparing normal and tumor tissues at the gene, mRNA, and protein levels. For example, integrated exome sequence data, DNA copy number, promoter methylation status, as well as mRNA and microRNA levels have been used to differentiate hypermutated from non-hypermutated tumors (8). Gene expression data from 4151 tumor specimens were used to classify CRC into four consensus molecular sequences (CMSs): CMS1 (immune features, microsatellite instability), CMS2 (canonical features), CMS3 (metabolic features), and CMS4 (mesenchymal features), in addition to subtypes characterized by mixtures of transition features (9). A study of transcriptomic differences between primary and metastatic tumors found that gene expression signatures specific to metastasis of colorectal adenocarcinoma were associated with reduced epithelial-mesenchymal transition (EMT) and increased activity of MYC-targeted and DNA repair pathways (10). This study also identified two CMSs associated with EMT-induced inflammation and proliferation in cancer metastases (10). However, the CMSs associated with adenoma remain unclear. CMS3 is the most common molecular subtype in adenoma, whereas CMS2 is the predominant subtype in adenomatous polyps, and CMS1 is highly associated with serrated polyps (11, 12). Despite the identification of various differentially expressed genes (DEGs) in CRC, the specific expression patterns of these genes during the onset of CRC remain unknown. Because of the limited number of studies, there are relatively few biomarkers for the individualized treatment of CRC. Moreover, the mechanisms underlying the onset and progression of CRC are not fully understood. Therefore, it is very important to identify the expression patterns of genes associated with the onset and progression of CRC.

In the present study, analysis of the transcriptomic profiles of primary CRC, adenoma, and normal colon epithelial tissues identified six specific dynamic expression patterns during tumor formation. Three dynamic patterns were associated with the three known cancer hallmarks (deregulation of cellular metabolism, avoidance of immune destruction, and activation of cancer associated pathway). Characterization of one pattern of six continuously and monotonically upregulated genes showed that specific clusters were negatively correlated with normal colon tissue and should be monitored during tumor formation. Among the genes in these patterns, *TPD52L1* was further screened as a poor prognostic marker in colon cancer that is associated with a low overall survival (OS) rate (*p* = 0.001), high recurrence rate (*p* = 0.0032), and a partial response to current therapeutic regimens. Furthermore, *TPD52L1* expression levels affect the malignant behavior of colon cancer cell lines by binding to its partners.

## Materials and Methods

### Antibodies and Primers

Antibodies against *TPD52L1*(hD53) (Proteintech, Wuhan, China, cat no. 14732-1-AP, 1:1000); and β-actin (Proteintech, cat no. 66009-1-Ig, 1:10000) were used for Western blot analysis. Magnetic beads against FLAG (Bimake, Houston, TX, USA, cat no. B26101, 1:10) were purchased for immunoprecipitation analysis. Antibodies against FLAG (TransGen Biotech Co., Ltd., Beijing, China, HT201, 1:200), anti-mouse Fluor 647 (Jackson ImmunoResearch Laboratories, West Grove, PA, USA, 1:600), and anti-rabbit Fluor 594 (Jackson ImmunoResearch Laboratories, 1:600) were used for immunofluorescence analysis. For quantitative real-time PCR (qRT-PCR), primers against qTPD52L1-U1 (TTT TGT CAG CGA AAG AAA GGC A) and qTPD52L1-D1 (GCA GTG GTA GTC TGC ATG TCA) were purchased from TransGen Biotech Co., Ltd., and primers against qTPD52L-U2 (PrimerBank ID: 51173743c1; AAC CGT TGC AAG GAA CAG AC), qTPD52L-D2 (ATG CCA GCT TTT GCT GAA GT) (13), hGAPDH-U (AGA AGG CTG GGG CTC ATT TG), and hGAPDH-R (AGG GGC CAT CCA CAG TCT TC) (14) were obtained from PrimerBank (https://pga.mgh.harvard.edu/primerbank/index.html).

### Cell Lines

The human colon cancer cell lines HT29, HCT116, RKO, and FHC were purchased from the Chinese Academy of Sciences (Shanghai, China) and cultured in Myco5A, Dulbecco’s modified Eagle’s medium, and RPMI 1640 medium (Gibco; Thermo Fisher Scientific, Inc., Waltham, MA, USA) supplemented with 10% fetal bovine serum (FBS; 16000-044; Thermo Fisher Scientific, Waltham, MA, USA) and 100 IU/ml of penicillin/streptomycin (15140-122; Thermo Fisher Scientific) under sterile conditions and maintained at 37°C in an incubator under an atmosphere of 5% CO_2_/95% air.

### Patients and Tissue Samples

All subjects signed informed consent forms before participating in the study. The study protocol was approved by the Ethics Committee of Zhongshan Hospital affiliated with the Shanghai Medical College of Fudan University (Project identification code: Y17-244) and conducted in accordance with the Declaration of Helsinki. Triplicate tissue samples (primary CRC, adjacent normal tissue, and adenoma; n = 15) were collected from five patients with CRC prior to any therapy at Zhongshan Hospital, and stored in RNAlater™ Stabilization Solution (AM7020; Thermo Fisher Scientific) for later analysis. The clinical data of the five patients are listed in **Table S1**. Primary CRC and matched non-tumor tissues from 95 patients were collected and fixed with 4% paraformaldehyde.

### RNA-Seq and Data Analysis

Total RNA samples were extracted with TRIzol™ Reagent (Thermo Fisher Scientific). Ribosomal-RNA-depleted and strand-specific libraries were constructed with the Ribo-Zero Gold Kit (Illumina, Inc., San Diego, CA, USA) and TruSeq Stranded Total RNA Sample Prep Kit (Illumina, Inc.), and sequenced using the HiSeq 2500 Sequencing System (Illumina, Inc.) by Genergy BioTech Co., Ltd. (Delhi, India). Cleaned RNAseq data were aligned to the human GRCh38 reference assembly using the HISAT2 alignment program (15). Only uniquely mapped reads were retained for reads counting with HTseq (16). DEGs were identified with the R package DESeq2 (17). To identify the DEGs between two tissue specimens in different transitional stages, the Wald test in DESeq2 was applied with a false discovery rate (FDR) of <0.05 and absolute log2 (fold change) of >1. To identify DEGs in all three tissue types, the likelihood-ratio test (LRT) was used with an FDR threshold of <0.05.

### Consensus Molecular Subtype Analysis

To identify the CMSs of adenoma and carcinoma, the raw count data of RNAseq of colon adenocarcinoma (COAD) were downloaded from The Cancer Genome Atlas (TCGA). Combined with the raw counts data generated by HTseq, the batch effects of the RNAseq were removed by the ComBat-seq method using the R package sva (18). For adenomas, the CMSs were identified using the random forests classifier. For carcinomas, the CMSs were identified using the single sample prediction classifier.

### Gene Set Enrichment Analysis

Gene Set Enrichment Analysis (GESA) was performed using GSEA software version 3.0 (https://www.gsea-msigdb.org/gsea/index.jsp) with the Molecular Signature Database gene set version 6.2 (19). Phenotype permutations were performed on all gene expression data with a permutation number of 1000. Pathways corresponding to genes enriched at the top or bottom of the gene sets were identified using the FDR value threshold (*p* < 0.05) and ranked according to the normalized enrichment score. Pathways associated with gene sets of interest were selected as described previously (9). Detailed information is provided below and in **Table S5**.

Canonical pathways included MAPK (Gene Ontology [GO], biological process [BP]), SRC, JAK-STAT (BioCarta database), proteasome (Kyoto Encyclopedia of Genes and Genomes [KEGG]), Notch, cell cycle, translation, integrin-β3, Wnt, EMT, TGF-β, and MYC (Reactome Pathway Database). Immune activation pathways included immune response (GO BP), PD1 signaling, leukocyte-mediated cytotoxicity and T helper cells, infiltration with natural killer cells and activation of T helper 17 cells, regulatory T cells (ImmuneSigDB) (20). Metabolic activation pathways included sugar, amino acid, nucleotide, starch, sucrose, glutathione, tyrosine, lysophospholipid, fatty acid, arachnoid acid, linoleic acid (KEGG), glutamine (GO BP), and lysophospholipid (PID).

### Dynamic Expression Model Analysis

Fragments per kilobase of transcripts per million mapped reads (FPKM) of DEGs identified with the LRT with an adjusted p value of < 0.05 were used for c-means clustering with the R package Mfuzz to characterize dynamic changes in expression patterns (21, 22). The Fuzzy c-means clustering is a soft clustering method performed with the Mfuzz algorithm with two key parameters (c = number of clusters and m = fuzzification parameter). The algorithm iteratively assigns the profile to the cluster with the shortest Euclidean distance while minimizing any objective function. In this study, the data were clustered with the parameters c = 6, m = 3.637426, and iter.max = 100.

### Weighted Gene Co-Expression Network Analysis (WGCNA)

Only samples with an RNA expression level ≥0.1 FPKM were retained. FPKMs of primary CRC tissues, adjacent normal tissues, and adenoma tissues were prepared by log2-transformation. A β value = 10 was used as the soft power empirically. A signed network of the Pearson correlation coefficients was constructed to generate an adjacency matrix with the parameter minModuleSize = 50, which indicates that no more than 50 genes are assigned to a single module (23). Modules were generated based on the topological overlap of the adjacency matrix, then clustered by average linkage hierarchical clustering. Finally, the modules were merged using the dynamic tree cut function implemented in the R package (24) with mergeCutHeight = 0.25. This analysis yielded 66 modules with a median of approximately 150 genes.

### OS Analysis and Disease-Free Survival Analysis

Clinical information and RNAseq data of CRC from TCGA database (http://gdac.broadinstitute.org/) were download with the command firehose_get. Tissue specimens were assigned to a high or a low expression group according to the median gene expression level. Kaplan–Meier analysis, the log-rank test, and Cox hazard regression analysis were applied to the 771 genes in cluster 6 using the R package survival (25). The R package survminer (26) was used for graphing OS and DFS rates associated with *TPD52L1* gene expression.

### Knockdown and Overexpression Experiments

Short hairpin RNA (shRNA)-based RNAi experiments were performed as described previously (27) using the following three *TPD52L1* shRNA oligonucleotide sequences and one scrambled control sequence: 1#control (ACT CGA CAC TAT AGT ATC TCA); 2#shTPD52L1 (gcA GAG TTA GTT CAG CTA GAA); 3#shTPD52L1 (ctC GGC ATG AAC CTG ATG AAT); and 4#shTPD52L1 (taG GCG GTA CGA ACC CTA ATG). All sequences were cloned into the pLKO.1-puro cloning vector. The full-length human *TPD52L1* cDNA sequence (NM_001318903) was cloned into the GV492-Ubi-MCS-3FLAG-CBh-gcGFP-IRES-puromycin plasmid, which was obtained from Shanghai GenePharma Co., Ltd. All constructs were verified by DNA sequencing.

HCT116 cells were transfected with shRNAs and RKO cells were transfected with expression vectors using lentivirus-based systems for 5 days. The transfection efficacy was validated by qRT-PCR and Western blot analysis. All assays were performed in triplicate.

### Cell Proliferation Assay

Cell proliferation was assessed using the Cell Counting Kit-8 (Dojindo Laboratories Co., Ltd., Kumamoto, Japan) in accordance with the manufacturer’s instructions. Briefly, 2,000 cells of each group were seeded into five wells of 96-well plates and incubated with assay buffer for 1 h. Absorbance at 450 nm was measured with a Universal Microplate Reader (BioTek Instruments, Inc., Winooski, VT, USA).

### Colony Formation Assay

For the colony formation assay, 1,000 cells were seeded in a 6-cm dish and cultured for at least 8 days at 37°C in an incubator with 5% CO_2_. The medium was refreshed every 3 days. Cell colonies were then fixed with 4% paraformaldehyde for 30 min at room temperature and stained with 1% crystal violet. The cell colonies were photographed and counted using Image J software (https://imagej.nih.gov/ij/).

### Wound Healing Scratch Assay

The wound healing scratch assay was used to compare the migratory capabilities of the different cell lines. Briefly, different groups of cells were seeded into triplicate wells of six-well plates (2 × 10^6^/well) in complete culture medium and cultured until confluent. Then, an artificial scratch wound was drawn at the center of each well and the culture medium was replaced with fresh culture medium supplemented with 2% serum. Photographs of the same locations were recorded at 0, 24, 48, and 72 h. The extent of cell migration was evaluated with Image J software.

### Transwell Invasion Assay

The cell invasion capability was assessed using the Transwell assay with 24-well invasion chambers and 8-μm pore inserts (Corning Incorporated, Corning, NY, USA). Briefly, the upper chambers were precoated with 50 µl of diluted Matrigel™ Basement Membrane Matrix (BD Biosciences, Franklin Lakes, NJ, USA) for 4 h at 37°C. Then, 5 × 105 cells were seeded into the upper chambers, which contained 200 µl of culture medium supplemented with 2% FBS, whereas the lower chambers contained 600 µl of complete medium supplemented with 10% FBS. After 24 h of incubation, cells that migrated through the Matrigel matrix and adhered to the reverse side of the upper chamber were fixed with 4% paraformaldehyde for 30 min at room temperature, and stained with 1% crystal violet. Cells in three random fields were photographed and counted using Image J software.

### GO and KEGG Analyses

Genes with dynamic expression patterns were subjected to GO analysis (https://david.ncifcrf.gov/GO) using the Database for Annotation, Visualization and Integrated Discovery (https://david.ncifcrf.gov/) and KEGG (https://www.genome.jp/kegg/) to search for BPs or main functions and signaling pathways with default parameters, respectively (28, 29), of DEGs and immunoprecipitated proteins. Only the top 10 enriched GO terms of the BP category and signaling pathways with *p* < 0.05 were reported.

### Mass Spectrometry-Based Immuno-Precipitation Proteomics

The binding partners of the hD53 protein were captured by IP and identified by MS (30). Different groups of cells were cultured in 15-cm dishes with three biological replicates. Then, 80 × 10^6^ cells per sample were collected and lysed in lysis buffer on the ice for 10 min. After centrifugation, the pellet was discarded and cells from different groups were incubated with anti‐FLAG magnetic beads overnight at 4°C with gentle rotation. The IP complex was washed five times with lysis buffer and once with ice‐cold phosphate-buffered saline (PBS). Subsequently, the protein samples were resolved by sodium dodecyl sulfate polyacrylamide gel electrophoresis. Following in‐gel staining with Coomassie blue, the whole gel lane was extracted and subjected to MS analysis. LC-MS/MS analyses were performed on a Orbitrap Fusion mass spectrometer (Thermo Fisher Scientific). The raw MS data files were extracted with Proteome Discoverer software (Thermo Fisher Scientific, version 2.4.0.305). All MS/MS samples were analyzed using the Sequest HT search engine in the SwissProt database (Taxonomy: Homo sapiens, 20231 entries). The Minora algorithm was used to perform label-free quantification. Only unique peptides were used for quantification of proteins with at least one unique peptide, and the method of normalization on protein median was used to correct experimental bias.

### Flow Cytometry Analysis

For cell cycle analysis, 5 × 10^5^ cells per sample were collected, rinsed twice with ice-cold PBS solution and fixed in chilled 70% ethanol at 4°C overnight. The fixed cells were washed with ice-cold PBS, incubated in 500 μl of staining buffer with 10 μl of RNase A and 25 μl of propidium iodide (Beyotime Biotechnology) in the dark at room temperature for 30 min. Stained samples were assessed with a FACS Aria II flow cytometer (BD Biosciences, San Jose, CA, USA) and data were analyzed using Flowjo software (version 7.6.1).

### Immunofluorescence Staining

Cells were fixed with 4% paraformaldehyde on glass coverslips and incubated with primary antibodies against hD53 and FLAG at 4°C overnight, washed with PBS, incubated with fluorescence-conjugated secondary antibodies (Jackson ImmunoResearch Laboratories), counterstained with anti-fade 4′,6-diamidino-2-phenylindole solution (Life Technologies, Carlsbad, CA, USA), and imaged using a confocal laser scanning microscope (882 SP5; Leica Microsystems GmbH, Wetzlar, Germany) with the same parameters for all samples.

### Immunohistochemical Analysis

Human tumor samples and paired noncancerous matched tissues were obtained from 95 CRC patients treated at Zhongshan Hospital. Written informed consent was obtained from each patient and the investigation was approved by the institutional review board of Zhongshan Hospital, Fudan University, Shanghai, China. IHC analysis of 95 pairs of tumors and matched peritumoral specimens was performed using a standard streptavidin biotin-peroxidase complex method. Paraffin-embedded, formalin-fixed sections were dewaxed, blocked with hydrogen peroxide/methanol solution, deparaffinized in xylene, and rehydrated in a decreasing ethanol series diluted in distilled water. After antigen retrieval with 8 mM citrate buffer under pressure, the slides were incubated overnight at 4°C with antibodies against hD53. Following 30 min of incubation with the secondary antibodies, the slides were developed in 3,3′-diaminobenzidine solution under microscopic observation and counterstained with hematoxylin. The extent of staining was scored on a scale of 0 to 4 corresponding to the percentage of immunoreactive tumor cells as follows: 0 points, 0%; 1 point, 1% to 5%; 2 points, 6% to 25%; 3 points, 26% to 75%; and 4 points, 76% to 100%. The staining intensity was scored as negative (score = 0), weak (score = 1), medium (score = 2), or strong (score = 3). A score ranging from 0 to 12 was calculated by multiplying the staining extent score by the intensity score. Two independent pathologists blinded to the patient outcomes scored the specimens.

### Statistical Analysis

All experiments were performed in triplicate and data are expressed as the mean ± standard deviation. Statistical analyses were performed using IBM SPSS Statistics for Windows, version 19.0. (IBM Corporation, Armonk, NY, USA) and GraphPad Prism 7.0 software (GraphPad Software, Inc., San Diego, CA, USA). Cumulative survival time was calculated by the Kaplan–Meier method and analyzed with the log-rank test. Univariate and multivariate analyses were based on the Cox proportional hazards regression model. The Pearson χ^2^ test and Student’s *t*-test were used for comparisons between groups. All data are presented as the mean ± standard deviation of three independent experiments. A probability (*p*) value less than 0.05 was considered statistically significant (*p < 0.05; **p < 0.01; **p < 0.001).

## Results

### Clinical Information of the Studied CRC Patients

CRC arises through a stepwise progression from normal colon epithelial tissue to adenoma and then to CRC. Triplicate tissue samples (primary CRC, adjacent normal tissue, and adenoma; n = 15) were collected from five patients with CRC prior to treatment. The expression profiles of target genes during CRC formation were assessed by RNAseq. The clinical characteristics of the five patients are summarized in **Table S1**. Basic information of RNA sequencing is summarized in **Table S2**. Because CRC is a heterogeneous disease, recurrence cannot be accurately predicted based solely on the tumor-node-metastasis staging system (31). Therefore, a comprehensive molecular classification system was established to stratify CRC patients according to the expression profiles of target genes. The results of the CMS classifier showed that all adenomas belonged to CMS3 regardless of the use of the random forests algorithm or the single sample prediction classifier, and they developed into different carcinoma CMSs (**Table S1**). The results of pathological analysis indicated that all collected adenomas were either tubular or tubulovillous with low-grade intraepithelial neoplasia (**Table S1**). Only 10% of polyps develop into carcinoma. Therefore, an adenoma in patients with malignancy as opposed to those without malignancy has greater potential to differentiate into an adenocarcinoma (32). All adenoma specimens in the present study were from patients with malignancy. GSEA of all genes between any two of the three tissue types was performed in reference to the GRADE_COLON_AND_RECTAL_CANCER_ UP data set to determine the malignancy status of adenoma based on significantly enriched genes (**Figure S1**).

### Identification of DEGs During CRC Formation

**Figure 1:**
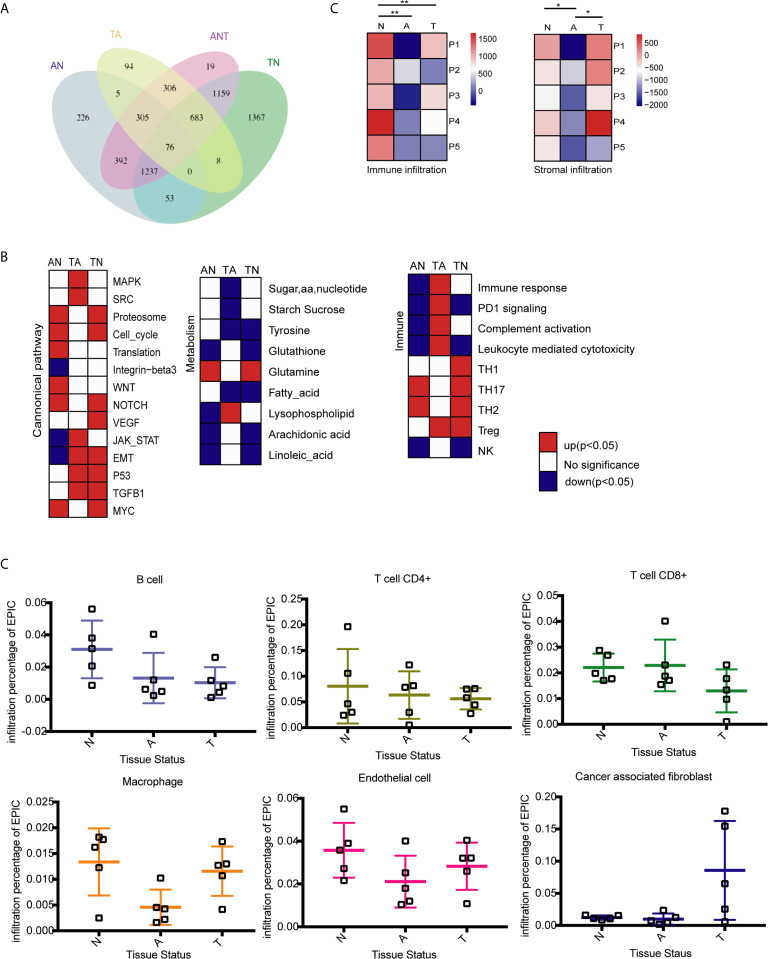
Identification of differentially expressed genes (DEGs) and molecular characteristics of colorectal cancer (CRC) formation by comparison of the transcriptome profiles at three stages of tumor formation. N, normal tissue; A, adenoma tissue; T, tumor tissue; AN, adenoma *vs.* normal colon tissues; TA, primary cancer *vs.* adenoma tissues; TN, normal colon *vs.* malignant tissues; ANT, three tissue types. **(A)** Venn diagram showing the overlap of DEGs identified by AN (blue), TA (yellow), TN (green), and ANT (purple) comparisons. **(B)** GSEA heatmap showing significant changes in the comparison of two stages of CRC formation with canonical pathway activity, metabolism, and immune processes data set. **(C)** Immune and stromal infiltration status of each sample, **P < 0.01, *P < 0.05, Student’s *t*-test. **(D)** Infiltration percentage of B cells, CD4+ T cells, CD8+ T cells, macrophages, endothelial cells and cancer associated fibroblasts in each sample by EPIC.

DEGs between two tissue types were identified using an FDR < 0.05 and absolute log2 (fold change) > 1. In total, 2294 DEGs were identified by comparing adenoma with normal colon tissues (AN), whereas 1477 DEGs were identified by comparing primary tumors with adenoma tissues (TA), suggesting that adenoma is more similar to cancerous tissue than to normal tissue (**Table S3**). Further comparisons of normal colon vs. primary tumor tissues (TN) identified 4585 DEGs (**Table S3**). Comparisons of all three tissue types (ANT) by LRT (DEseq2) identified 4177 DEGs with FDR-adjusted *p* values less than 0.05 (**Table S4**). The overlapping DEGs between normal colon, adenoma, and primary tumor tissues are shown in **Figure 1A**. Although comparisons between any two tissue types identified DEGs showing relative upregulation or downregulation, changes in expression patterns during the adenoma-carcinoma sequence were difficult to determine.

### Early and Late Stage of CRC Onset Are Associated With Three Hallmarks

Reprogramming energy metabolism and evading immune detection are important biological properties of cancer cells (33). Early CRC research indicated that mutations to the APC gene that drive the upregulation of the Wnt pathway are an initiating event that gives rise to the adenoma-carcinoma sequence (34). However, the roles of other cancer-associated pathways in tumor onset remain unclear. Changes to pathways associated with metabolism and the immune response have been characterized in different CRC subtypes (9, 33), whereas dynamic patterns characteristic of the adenoma-carcinoma sequence remain unknown. GSEA was performed to compare the transcriptome profiles of any two of the three tissue types. Previously reported gene sets involved in canonical pathway activities and metabolic and immune processes were analyzed (9, 33), and enriched genes sets (*p* < 0.05) were selected to generate heatmaps (**Figure 1B**).

The stages of tumor formation are generally classified as early stage (normal to adenoma transition) or late stage (adenoma to carcinoma transition). During the transition from normal colon epithelium to adenoma and then to carcinoma, most canonical pathways were activated, but only the integrin-β3, Jak-stat, and EMT pathways were downregulated during normal to adenoma transition. The proteasome, cell cycle, and translation activities, as well as the Wnt, NOTCH, and Myc pathways were activated early during normal to adenoma transition, whereas the MAPK, Src, JAK-STAT, EMT, p53, and TGFB1 pathways were activated late during adenoma to carcinomas transition (**Figure 1B** and **Table S5**).

Dysregulation of metabolic activity is a characteristic of tumor formation. GSEA revealed that the activities of most metabolism-associated pathways were decreased during tumor formation. Notably, glutamine anaplerosis was increased during normal to adenoma transition, whereas lysophospholipid metabolism was increased during adenoma to carcinoma transition (**Figure 1B** and **Table S5**). Glutamine is a major anaplerotic substrate in the tricarboxylic acid cycle and a feature of the metabolism of cancer cells (35). Lysophospholipids, including lysophosphatidic acid and sphingosine-1-phosphate, are major mediators of intercellular communication involved in the regulation of tumor cell growth and the modulation of the immune response (36).

The immune system plays important roles in tumor onset and progression. Immune responses associated with tumoricidal activities, including complement activation, leukocyte-mediated cytotoxicity, and the activation of natural killer cells, were repressed during normal to adenoma transition and activated during adenoma to carcinoma transition (**Figure 1B** and **Table S5**). By contrast, regulatory immune pathways, except PD1 signaling, were activated, including T helper 2 and 17 cells in normal to adenoma transition and regulatory T cells in adenoma to carcinoma transition.

To determine the effect of the tumor microenvironment, the infiltration of immune cells and stromal cells in the three tissue types was investigated using the ESTIMATE algorithm (37) Immune cell infiltration was significantly lower in adenomas and carcinomas than in normal tissues, whereas stromal cell infiltration was lower in adenomas than in normal and carcinoma tissues (**Figure 1C**). Analysis of infiltrating immune cells and stromal cells in the different tissues showed that the percentage of B and CD4+ T cells was decreased during tumor formation, the percentage of CD8+ T cells was decreased in malignant tissues, and macrophage infiltration was decreased in adenoma tissues but increased in malignant tissues (**Figure 1D**).

GSEA revealed that changes in canonical pathways, metabolism, and immune responses were not consistent during the normal adenoma-carcinoma sequence. Some changes were initiated during normal to adenoma transition, whereas others were triggered during adenoma to carcinoma transition, and there were various changes to some pathways in different transitions. To clarify these results, we further explored the expression patterns of DEGs during tumor formation.

### Identification of Dynamic Expression Patterns in the Adenoma-Carcinoma Sequence of CRC

**Figure 2:**
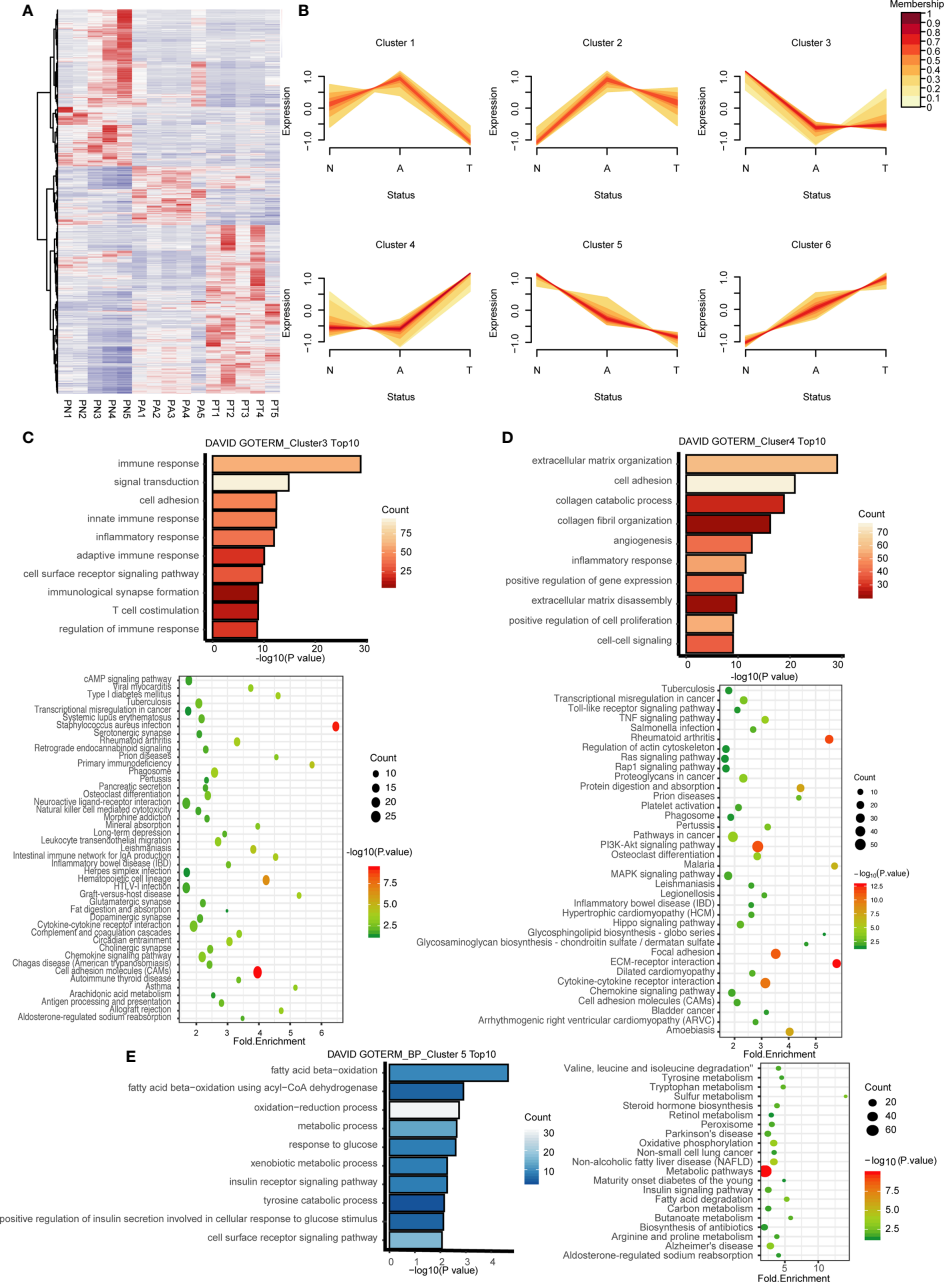
Identification of dynamic expression patterns and molecular characteristics of 4177 DEGs from the ANT comparison. **(A)** Heatmap and hierarchical clustering of 4177 DEGs from the ANT comparison. PN, normal patient tissues; PA, adenomas tissues; PT, carcinoma tissues. **(B)** The series of diagrams illustrates the patterns of dynamic changes in DEGs during the formation of CRC determined using Mfuzz. **(C)** GO analysis of biologic processes and KEGG analysis of the genes in cluster 3. **(D)** GO analysis of biologic processes and KEGG analysis of the genes in cluster 4. **(E)** GO analysis of biologic processes and KEGG analysis of the genes in cluster 5.

To understand the dynamic changes of DEGs at three stages of CRC formation, unsupervised hierarchical clustering was performed based on the DEGs identified by comparisons of the three tissue types, and a heatmap of 4,177 DEGs in the 15 samples was created. As shown in **Figure 2A**, clusters of genes with different expression levels were enriched in different tissue types. To analyze the dynamic expression patterns of DEGs during the onset of CRC, the 4177 DEGs were classified into six patterns (clusters 1 to 6) using Mfuzz (**Figure 2B**, **Table S6**). The expression patterns of genes in clusters 1 and 4 changed significantly in the late period of tumor formation, whereas up- or downregulation of DEGs occurred only during adenoma to malignant transition. The expression patterns of DEGs in clusters 2 and 3 changed significantly in the early stage of tumor formation, whereas up- or downregulation of DEGs occurred only during normal to adenoma transition. DEGs in clusters 5 and 6 were continuously and monotonically downregulated or upregulated during the transition from normal tissue to malignant tissue (**Figure 2B**). The relationships between the six dynamic expression patterns and canonical pathways, metabolism, and immune responses remain to be elucidated. To validate the expression patterns existing universally in the adenoma-carcinoma sequence, patient-matched microarray data (GSE117606) were soft clustered into 9 expression patterns which contained the six specific patterns identified by our analysis above (**Figure S2**).

### Pathways Associated With Dynamic Expression Patterns

We hypothesized that genes with different expression patterns have different functions during CRC formation. GO and KEGG analyses of the six clusters revealed unique characteristics of genes in three of the six clusters. Genes in cluster 3, which were downregulated during normal to adenoma transition, were mainly associated with the immune response (GO terms included innate immune response, inflammatory response, adaptive immune response, immunological synapse formation, and regulation of immune response; **Figure 2C**). KEGG analysis indicated that genes in cluster 3 were involved in pathways associated with the immune response, especially innate immunity, including infectious immunity, primary immunodeficiency, natural killer cell-mediated cytotoxicity, the complement and coagulation cascades, and graft-versus-host disease, among others (**Figure 2C**). Genes in cluster 4, which were upregulated during adenoma to carcinoma transition, were mainly involved in canonical pathways associated with cancer, including the TNF, Toll-like receptor, Ras, MAPK, and Hippo signaling pathways, among others (**Figure 2D**). GO analysis revealed that genes in cluster 5, which were monotonously downregulated during the adenoma-carcinoma sequence, were associated with the BPs of metabolism, including beta oxidation of fatty acids, the oxidation−reduction process, response to glucose, xenobiotic metabolism, the insulin receptor signaling pathway, tyrosine catabolism, and the cell surface receptor signaling pathway (**Figure 2E**). Similar results were obtained by KEGG analysis, which showed that genes were mainly involved in metabolic pathways, including tryptophan metabolism, sulfur metabolism, steroid hormone biosynthesis, oxidative phosphorylation, non-alcoholic fatty liver disease, the insulin signaling pathway, fatty acid degradation, and butanoate metabolism (**Figure 2E**). The results of GO and KEGG analyses of the other clusters are shown in **Figure S3**.

The biologic characteristics of the three clusters were validated with the GSE117606 data set. The genes associated with immune response (*TNFRSF1A*, *CMKLR1*, *CTSG*, *CD22*, *SECTM1*) had similar expression patterns in cluster 3 (**Figure S4A**), while those associated with the canonical cancer pathway (*MMP14*, *NOTCH3*, *TGFB3*, *TGFBR1*, *VEGFA*) share expression patterns with cluster 4 (**Figure S4B**) and those associated with metabolism (*ACACB*, *GALNT12*, *MAOA*, *PLCD1*, *SMPD3*) had similar expression patterns in cluster 5 (**Figure S4C**).

### Dynamic Expression Patterns Associated With Pathological Phenotype in the Adenoma-Carcinoma Sequence

**Figure 3:**
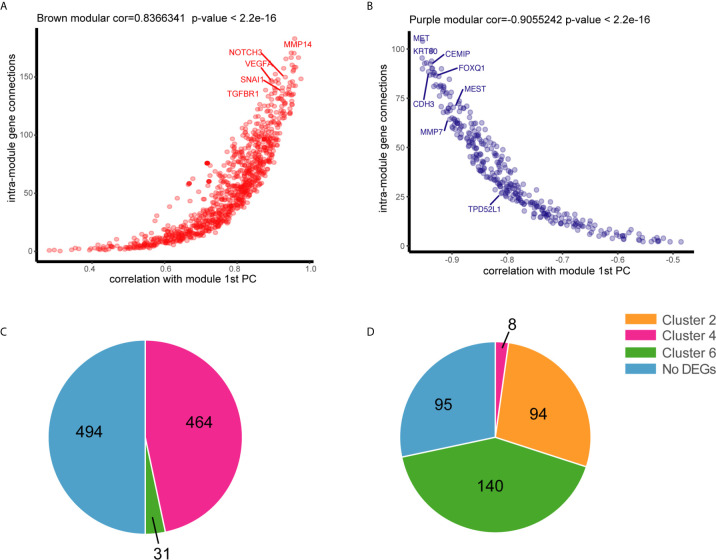
Association of genes with tissue status by weighted gene co-expression network analysis WGCNA. **(A)** Connectivity between genes within the purple module is plotted against the module’s first principal component (module gene expression). **(B)** Connectivity between genes within the brown module is plotted against the module’s first principal component. **(C)** Distribution of positive genes in the brown module in six dynamic expression patterns. **(D)** Distribution of negative genes in the purple module in six dynamic expression patterns.

To understand the relationship between DEGs and the pathology of tissues, WGCNA was applied to 32,682 genes with an expression of at least 0.1 FPKM, which yielded 66 modules of highly co-expressed genes with a median of approximately 150 genes. DEGs in two of the modules were highly associated with tissue phenotype. DEGs in the brown module were highly associated with malignancy (cor = 0.86, *p* = 4e-05), whereas those in the purple module were associated with normal tissues (cor = 0.94, *p* = 1e-07) (**Figure S5**). Most DEGs in the brown module were involved in tumor progression or metastasis (module membership >0, cor = 0.83, *p* < 2.2e-16), including *NOTCH3*, *VEGFA*, *SNAL1*, *TGFBR1*, and *MMP14* (**Figure 3A**), thereby confirming the correlation of some DEGs with phenotypes. DEGs in the purple module (module membership <0) were negatively correlated with normal tissues and positively associated with adenoma and malignant tissues. Several of these DEGs were associated with CRC, including *MET*, *CEMIP*, *KRT80*, *MMP7*, *MEST*, and *CDH3* (**Figure 3B**). DEGs in these two modules had dynamic expression patterns. DEGs in the brown module that were associated with malignant tissues were enriched in clusters 4 (464/989) and 6 (31/989) (**Figure 3C**). The genes that were not associated with normal tissues in the purple module were enriched in clusters 6 (140/337), 2 (94/337), and 4 (8/337) (**Figure 3D**). The DEGs in cluster 4 may be important for the ultimate deterioration of tissues, whereas those in cluster 6, which were associated with abnormal conditions, may be involved in key early events of tumor formation.

The functions of DEGs in cluster 6 remain unclear because of limited data. GO and KEGG analyses indicated that these DEGs were involved in relatively few pathways compared with those in other clusters. Nonetheless, KEGG analysis revealed that these DEGs were associated with three of four DNA damage repair pathways, including nucleotide excision repair, non-homologous end-joining, and base excision repair (**Figures S2C**). Repair of damaged DNA is crucial for tumor survival. Loss of DNA mismatch repair causes instability of colon cancer microsatellites, which is a known mechanism of CRC. The biological functions of the other three DNA repair pathways in CRC are unknown. DEGs in cluster 6 with continuous and monotonic expression patterns were not associated with normal tissues. However, the data on the BPs associated with these DEGs were insufficient.

### High Expression of *TPD52L1* Is Associated With a Poor Prognosis in CRC

**Figure 4:**
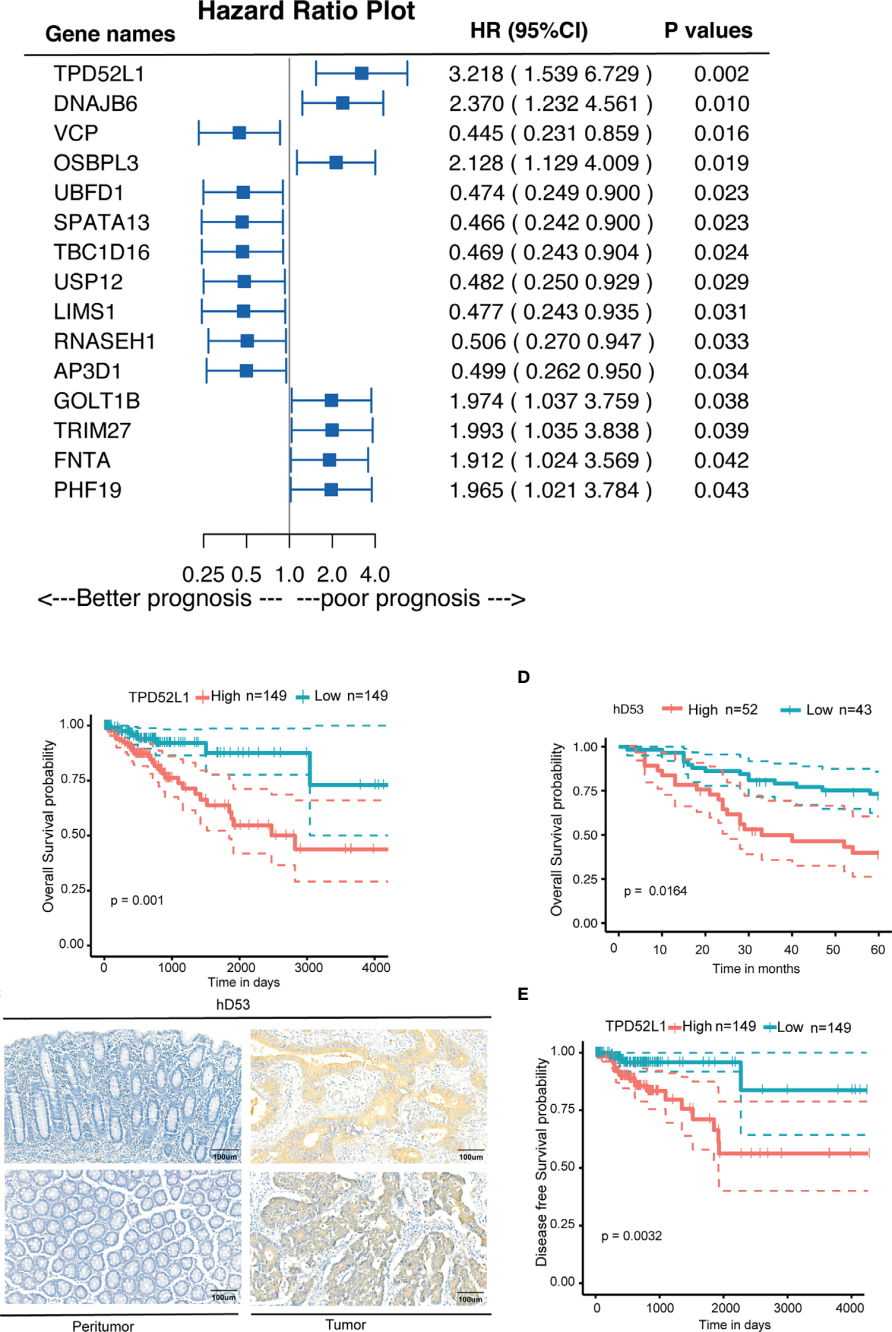
Clinical implications of TPD52L1 expression in CRC. **(A)** Forest diagram showing the hazard ratio and 95% confidence interval according to the Cox regression analysis of 16 genes in cluster 6 with p ≤0.05; **(B)** Kaplan-Meier survival curves with 95% confidence intervals (dashed lines) of OS stratified by low (149) vs. high TPD52L1 (149) mRNA levels in primary colon cancer tissues from TCGA data set. **(C)** Representative TPD52L expression in the normal colon epithelium and CRC tumor tissues detected by immunostaining with anti-TPD52L1 antibody (brown). The slide was counterstained with hematoxylin. Scale bar, 100 μm. **(D)** Kaplan-Meier survival curves of OS stratified by low (score<6, blue curve) vs. high (score ≥6, red curve) TPD52L1 expression levels (low, 43 patients; high, 52 patients) in primary colon cancer tissues from Zhongshan Hospital. **(E)** Kaplan-Meier survival curves with 95% confidence intervals (dashed lines) of disease-free survival stratified by low (149) vs. high (149) TPD52L1 mRNA levels in colon cancer tissues from TCGA data set.

Among the six dynamic expression patterns, the characteristics of genes in clusters 5 and 6 are usually concealed and the monotonous changes following the formation of CRC indicated that these genes may be suitable as specific tracking biomarkers or driving factors. DEGs in cluster 6 were selected for further analysis as markers of CRC formation. Kaplan–Meier analysis was performed to estimate the probability of OS and the log-rank test was used to compare groups with different expression levels of the 717 genes in cluster 6 based on TCGA data set (**Figure S6**). The 16 genes with p < 0.05 were analyzed using a Cox proportional hazard model, which showed that high expression levels of *TPD52L1* (hazard ratio [HR] = 3.218), *DNAJB6* (HR = 2.37), *OSBPL3* (HR = 2.128), *GOLT1B* (HR = 1.974), *TRIM27* (HR = 1.993), *PHF19* (HR = 1.965), and *FNTA* (HR = 1.912) were associated with a poor prognosis of CRC patients (**Figure 4A**). Seven genes were associated with phenotypes, as determined by WGCNA. *TPD52L1* was highly negatively correlated (−0.81) with DEGs in the purple module, which are associated with normal tissues, suggesting that *TPD52L1* is a potential marker of non-normal phenotypes involved in tumor formation (**Figure 3B**).

Although the clinical information of 485 patients from the COAD data set (TCGA) indicated that high mRNA level of TPD52L1 was associated with a lower OS rate (p = 0.001) and poor prognosis (**Figure 4B**). Tissues from 95 patients were collected and the expression levels of hD53, the protein encoded by *TPD52L1*, were assessed in primary CRC and matched non-tumor tissues by IHC (**Figure 4C**) to independently verify the prognostic significance of *TPD52L1*. The clinical characteristics of the patients are shown in **Table 1**. hD53 was seldom detected in the normal colon epithelium, whereas positive expression was detected in COAD tissues (**Figure 4C**). Expression of hD53 was detected in 95/95 (100%) of COAD tumor tissues compared with the corresponding peritumor tissues. Based on a median immune score of 6 as the cutoff value for hD53 staining, COAD tissues were divided into two groups: a high expression group (immune score ≥6, n = 52) and a low expression group (immune score <6, n = 43). The results of the chi-square test revealed that high hD53 expression was significantly associated with lymph node metastasis (*p* = 0.011), distant metastasis (*p* = 0.033), and advanced clinical stage (*p* = 0.009) (**Table 1**). Kaplan–Meier survival curve analysis indicated that high hD53 expression was associated with a lower OS rate (log-rank test, *p* < 0.05, **Figure 4D**). *TPD52L1* is downregulated in post-chemotherapy ovarian tumors (38) and in recurrent nasopharyngeal cancer after the first course of radiotherapy (39), suggesting that *TPD52L1* expression is associated with tumor recurrence and responses to current therapeutic drugs against CRC. DFS analysis of TCGA data indicated that high *TPD52L1* expression was associated with significantly earlier relapse (*p* = 0.0032) (**Figure 4E**).

**Table 1:** Clinicopathological correlation of *TPD52L1* high in CRC.

Clinical features	Cases	*TPD52L1* expression		P value
		Low group	High group	
		(%)	(%)	
Age (years old)				0.152
≤59	52	27 (51.9%)	25 (48.1%)	
>59	43	16 (37.2%)	27 (62.8%)	
Sex				0.752
Male	58	27 (46.6%)	31 (53.4%)	
Female	37	16 (43.2%)	21 (56.8%)	
Location				0.797
Right	30	13 (43.3%)	17 (56.7%)	
Left	65	30 (46.2%)	35 (53.8%)	
LN metastasis				**0.011**
N0	46	27 (58.7%)	19 (41.3%)	
N1	49	16 (32.7%)	33 (67.3%)	
Distant metastasis				**0.033**
M0	46	26 (56.5%)	20 (43.5%)	
M1	49	17 (34.7%)	32 (65.3%)	
Clinical stage				**0.009**
Early (I−II)	29	19 (65.5%)	10 (34.5%)	
Advanced (III−IV)	66	24 (36.4%)	42 (63.6%)	

Statistical significance (P < 0.05) is shown in bold emphasis.

We next analyzed whether *TPD52L1* is associated with the TNM stages of colon cancer. RNAseq data of TCGA database showed that expression of *TPD52L1* was higher in CRC tissues at all stages as compared to normal colon tissues (**Figure S7**), whereas there was no significant difference between TNM stages.

Taken together, these findings indicate that the *TPD52L1* gene is continuously and monotonically upregulated during tumor formation and highly associated with the non-normal phenotype, suggesting a potential role in tumor formation. High expression of *TPD52L1* is thus a potential biomarker associated with poor prognosis and earlier relapse in CRC.

### *TPD52L1* Promoted Oncologic Behavior of Cancer Cells

The 10 hallmarks of cancer, including sustained proliferative signaling, evasion of growth suppressors, resistance to cell death, enabling of replicative immortality, induction of angiogenesis, activation of invasion and metastasis, deregulation of cellular energetics and metabolism, and avoidance of immune destruction (33), provide a solid basis to understand the biology of cancer. To explore the role of *TPD52L1* in CRC, gain-of-function and loss-of-function studies were designed to detect the effects of *TPD52L1* on the oncologic behaviors of CRC including cell proliferation, migration, invasion, and colony formation ability.

To determine appropriate cell models for function studies of *TPD52L1*, the RNAseq data of seven common CRC cell lines from the Cancer Cell Line Encyclopedia were investigated (**Figure S6A**). The results showed that *TPD52L1* was expressed in all CRC cell lines at different levels. Expression of *TPD52L1* was validated in the cell lines in our lab by qRT-PCR (**Figure S8B**) and Western blotting (**Figure S8C**). FHC, a normal fetal epithelial cell line, did not express *TPD52L1* (delta ct >40, no band on Western blot), suggesting that *TPD52L1* is associated with CRC formation. Given that RKO cells have relatively low protein levels of hD53 and HCT116 cells are more metastatic than HT29, these cell lines were used for hD53 overexpression and knockdown studies, respectively.

**Figure 5:**
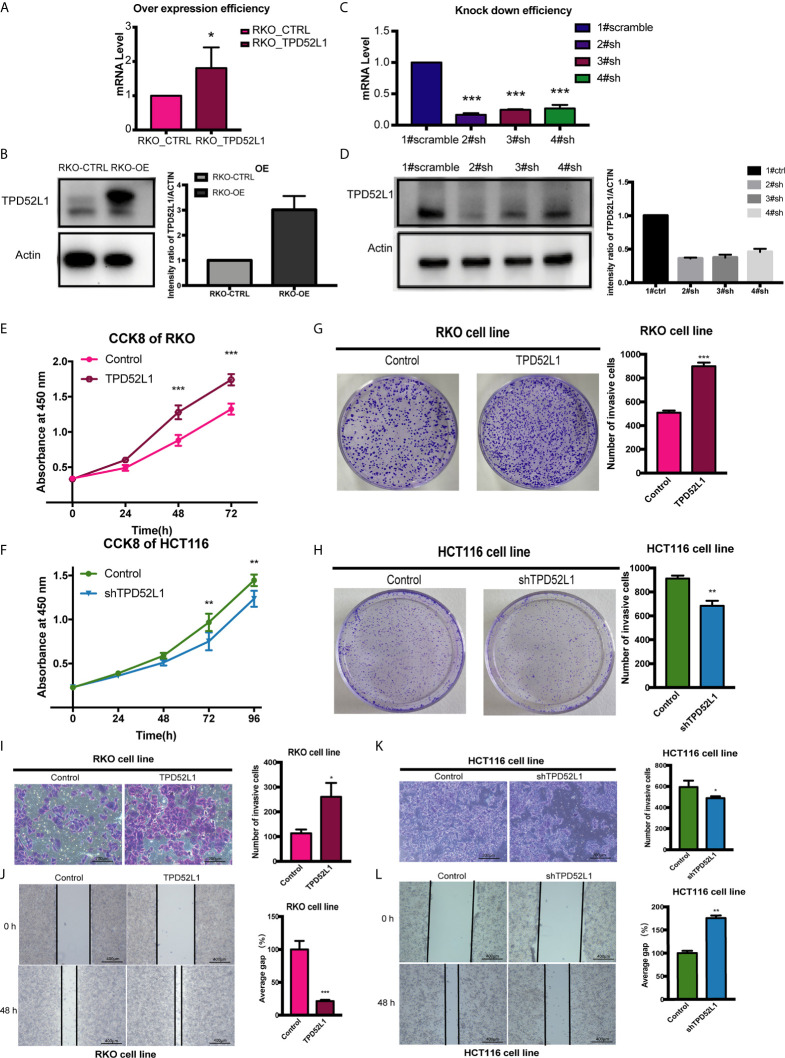
Effect of TPD52L1 on tumor behavior in *vitro*. **(A)** TPD52L1 mRNA levels detected by qRT-PCR in the RKO cell line (control and overexpression); GAPDH was used as negative and endogenous control, *P < 0.05, Student’s *t*-test. **(B)** Western blot analysis of TPD52L1 protein levels in RKO cells (control and overexpression). β-actin was used as the loading control. **(C)** Three shRNAs (2#sh, 3#sh, and 4#sh) against TPD52L1 effectively decreased TPD52L1 expression as detected by qRT-PCR. Negative control (1# scramble) and GAPDH were used as negative and endogenous controls, respectively, ***p < 0.001, Student’s *t*-test. **(D)** Three shRNAs (2#sh, 3#sh, and 4#sh) against TPD52L1 effectively decreased TPD52L1 expression as detected by Western blotting. Negative control (1# scramble) and β-actin were used as negative and endogenous controls, respectively. **(E)** TPD52L1 overexpression increased the proliferation ability of RKO cells, ***P < 0.001, Student’s *t*-test. **(F)** TPD52L1 knockdown decreased the proliferation ability of HCT116 cells, **p < 0.01, Student’s *t*-test. **(G)** TPD52L1 overexpression increased the colony formation ability of RKO cells, ***p < 0.001, Student’s *t*-test. **(H)** TPD52L1 knockdown suppressed the colony formation ability of HCT116 cells, **p < 0.01, Student’s *t*-test. **(I)** TPD52L1 overexpression increased the invasion ability of RKO cells, *p < 0.05, Student’s *t*-test. **(J)** TPD52L1 overexpression increased the migration ability of RKO cells, ***P < 0.001, Student’s *t*-test. **(K)** TPD52L1 knockdown decreased the invasion ability of HCT116 cells, *p < 0.05, Student’s *t*-test. **(L)** TPD52L1 knockdown decreased the migration ability of HCT116 cells, **p < 0.01, Student’s *t*-test.

hD53 fused with the FLAG protein was successfully expressed in RKO cells at both the mRNA (**Figure 5A**) and protein (**Figure 5B**) levels. Immunofluorescence staining showed that FLAG-tagged hD53 localized mainly to the cytoplasm, with a small amount expressed in the nucleus, which was consistent with the expression pattern of the endogenous protein (**Figure S9**). The transfection efficacy of three different shRNAs in HCT116 cells was assessed by qRT-PCR (**Figure 5C**) and Western blotting (**Figure 5D**). Among the three shRNAs, 2#shRNA showed the highest knockdown efficacy and was therefore selected for subsequent experiments in HCT116 cells.

The results of the cell proliferation assay demonstrated that lentiviral-mediated exogenous hD53 expression significantly promoted the growth of RKO cells (**Figure 5E**), whereas knockdown of endogenous hD53 by RNA interference inhibited HCT116 cell proliferation (**Figure 5F**). The results of the cell colony formation assay indicated that hD53 overexpression increased the colony formation abilities of RKO cells (**Figure 5G**), whereas hD53 downregulation decreased colony formation in HCT116 cells (**Figure 5H**). The results of cell migration and invasion assays showed that hD53 overexpression increased the migration and invasion abilities of RKO cells as compared with those of control cells (**Figures 5I, J**, respectively), whereas TPD52L1 knockdown had the opposite effect on the metastatic ability of HCT116 cells (**Figures 5K, L**).

### *TPD52L1*-Associated Proteins and Involved Pathways

**Figure 6:**
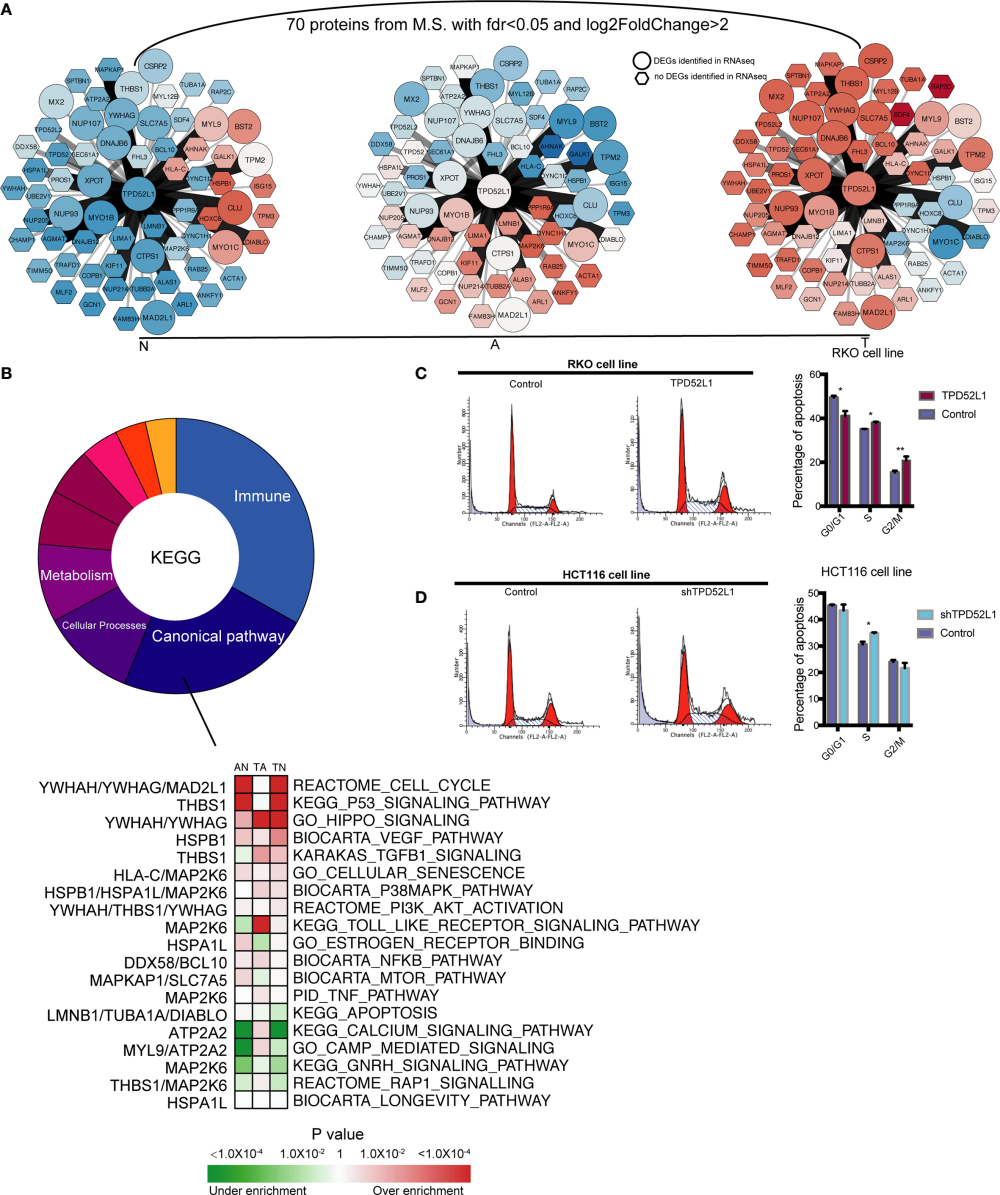
Identification the partners of TPD52L1 involved in CRC formation. **(A)** Changes to the expression level of the partners of TPD52L1 during CRC formation. Hexagon node shape: no DEG in RNAseq; Round node shape: no DEG in RNAseq. **(B)** Distribution of pathways of the partners of TPD52L1 involved in CRC formation. **(C)** TPD52L1 overexpression prompted cell cycle arrest in RKO cell line, *p < 0.05,**p < 0.01, Student’s t-test. **(D)** TPD52L1 knockdown arrested cell cycle in the S phase, *p < 0.05, Student’s t-test.

To explore the mechanism of TPD52L1 in cancer cell lines, the 70 proteins interacted with TPD52L1 were identified by comparing the proteomics results of IP from the TPD52L1-3×FLAG and 3×FLAG-only groups with FDR < 0.05 and log2 (fold change) > 2 (**Table S7**). Among the 70 proteins, members of the D52-like protein family (*TPD52*, *TPD52L2*, and *TPD52L1*) and members of the 14-3-3 protein family (*YWHAH* and *YWHAG*), which are known partners for hD53 (40–43). According to proteomics results, TPD52L1 and its 70 interacting proteins were constructed into a TPD52L1-centred network. In this network, the edges were weighted by log2 fold-change of abundance and the nodes were weighted according to the row-scaled transformed data of gene expression (FPKM) across the three time points during the tumor formation (**Figure 6A**).

Most of these 70 proteins were slightly up-regulated along with TPD52L1 during the formation of CRC, and the expression patterns of 10 differential genes are consistent with *TPD52L1* (*TPD52L1*, *DNAJB6*, *YWHAG*, *NUP07*, *NUP93*, *XPOT*, *MAD2L1*, *CTPS1*, *SLC7A5*, and *MYO1B*; **Figure 6A**). Because research of the hD53 protein is limited, the pathways of interacting proteins may offer hints of the mechanism of *TPD52L1*. KEGG analysis revealed that the 70 interacting proteins were involved in 110 pathways which were mainly associated with immunity (36/110), canonical pathways (25/110), cellular processes (12/110), and metabolism (10/110) (**Figure 6B**; **Table S8**). Among the canonical pathways, *YWHAG* was associated with the cell cycle, Hippo, and PI3K-AKT pathways. Based on above information, cell cycle analysis revealed that *TPD52L1* overexpression increased the number of RKO cells in the S and G2/M phases (**Figure 6C**), whereas *TPD52L1* knockdown in HCT116 cells caused cell cycle arrest in the S phase (**Figure 6D**). These results suggest that influencing the cell cycle by binding 14-3-3 proteins may be one of pathways that *TPD52L1* affects tumor behavior.

## Discussion

Early studies analyzing the cumulative mutation and activation of oncogenes, inactivation of tumor-suppressor genes, and deletion of genes associated with CRC carcinogenesis provided a partial interpretation of the genetic alterations that occur during CRC development (34, 44–46). Analysis of the transcriptome profiles during the mucosal adenoma-carcinoma sequence in CRC may shed new light on the mechanisms underlying the onset of CRC. A recent study identified 336 DEGs related to tumor formation at different stages of CRC, including seven that were associated with the prognosis of CRC patients (47). However, the results may have been influenced by differences in patient characteristics. RNAseq data obtained from patient-matched samples of the adenoma-carcinoma sequence in CRC, as performed in the present study, can reduce the effect of differences among patients by the LTR test. Heatmaps of the six dynamic expression patterns showed differences in patient characteristics had no effect on the clustering results (**Figure S10**). Although the sample size was limited because of the difficulty of collecting patients, the results were verified with the use of a public data set. This analysis clarified differences in the features of these genes between two tissue types, which are concealed by RNAseq analysis in other studies.

In the present study, all adenoma samples were classified as CMS3, consistent with an earlier study (9). The CMS3 classification is characterized by normal gene expression levels (11). Adenomas of the CMS3 subtype develop into tumors of different CMSs, implying that the molecular characteristics of CRC are acquired during adenoma to carcinoma transition. Among the six dynamic expression patterns, genes in cluster 4, which changed only during adenoma to carcinoma transition, were enriched in the canonical cancer pathways. Genes in cluster 4 associated with cancer tissue (0.83) included many oncogenes: NOTCH3 signaling promotes tumor growth in CRC (48), whereas MMP14 and VEGFA are correlated with poor prognosis (49, 50). Any gene following the dynamic expression patterns of cluster 4 during tumor formation may play a critical role in malignant transformation. This pattern may help reduce the workload of screening DEGs as new targets of malignant transition.

Our analysis of the tumor microenvironment showed that immune infiltration was decreased in the earlier stages of tumor formation, which is inconsistent with the results of IHC and qRT-PCR analyses of the pro-inflammatory environment of adenomas (51). However, immune infiltration of CRC tissues occurs in parallel with antagonistic suppression of pro-inflammatory immune responses (52, 53). Infiltration of CD4+ T cells is suppressed by Wnt signaling in CRC (54), whereas infiltration of CD8+ T cells decreases progressively and that of regulatory T cells increases gradually during CRC onset (55). The percentage of infiltrating macrophages decreased during tumor onset and increased during tumor progression (**Figure 1D**), which may explain the functional shift of macrophages from tumoricidal to tumorigenic activities (56). Alternatively, there could be two groups of macrophages. The percentages of infiltrating endothelial cells and cancer-associated fibroblasts were estimated. Fibroblasts, which are highly abundant and play a role in CRC, are divided into normal mucosal fibroblasts, cancer-associated fibroblasts, and metastasis-associated fibroblasts (57, 58). Fibroblasts in the normal colorectal mucosa evolve into carcinoma-associated fibroblasts during adenoma to carcinoma transition. GO and KEGG pathway analyses of DEGs in the six dynamic expression patterns revealed immune deregulation occurred early during the normal mucosa to adenoma transition. These immune pathways are mainly associated with innate immunity. Over the last decade, oncological immune studies have overlooked the contribution of innate immunity compared with that of adaptive immunity. Increasing evidence also suggests that innate immunity may play an important role in the carcinogenesis of intestinal cancers (59). Thus, harnessing the adaptive and innate immune systems is an important strategy for the treatment of CRC (60).

RNAseq analysis of a public database led to the selection of *TPD52L1* as a research target. The *TPD52L1* gene is located on chromosome 6q22-23 and consists of eight exons. Transcripts containing exon 6, which includes a 14-3-3 binding motif, are most widely expressed (40). The *TPD52L1* gene encodes the tumor protein D53, which was first identified in a human breast carcinoma cDNA library and shows 52% identity to hD52 (42). The soluble cytoplasmic protein hD53 consists of 204 amino acids and contains a coiled-coil domain, an overlapped PEST domain, and 14 phosphorylation sites (42).

The results of the present study suggest that *TPD52L1* tracking during CRC formation is clinically valuable. Clinical information from TCGA showed that high *TPD52L1* expression was associated with poor prognosis, a higher probability of tumor relapse, and a partial response to current therapeutic regimens (**Figure S11**). The reduced OS and DFS rates may be the result of a partial response to therapy; however, the variability in the responses suggests that current regimens for the treatment of CRC should be further scrutinized. FOLFOX (folinic acid, 5-FU, oxaliplatin) and FOLFIRI (folinic acid, 5-FU, irinotecan) are currently the two most common regimes for the treatment of CRC. A partial response to these two regimens may be due to one of the drugs alone or the combination of multiple drugs. The expression level of *TPD52L1* in tumor tissues has clinical significance as a marker of poor prognosis and relapse, as well as implications regarding the efficacy of anti-cancer drugs.

The involvement of *TPD52L1* in various cancers was reported previously. Significantly high *TPD52L1* transcript levels (*p* = 0.007) in primary breast cancers at the time of surgical removal is a potential biomarker of lymph node metastasis (61). Ulcerative colitis (UC) increases the risk for CRC, and *TPD52L1* expression increases progressively from controls, to nondysplastic UC, to UC with neoplasia (62). In a hepatocellular carcinoma study, *TPD52L1* was one of eight mRNA biomarkers identified as independent risk factors and was used to develop a prognostic model of OS (63). In non-small-cell lung carcinoma, patients with the *TPD52L1*-ROS1 fusion variant [i.e., fusion of the coiled-coil domain of *TPD52L1* and kinase domain of ROS1 (64)] could benefit from treatment regimens incorporating a ROS1 inhibitor (65).

Although further research is necessary to clarify the underlying mechanism, the partners of hD53 were identified by proteomics as an important complement to the limited research of *TPD52L1*. *YWHAG* is an oncogene that encodes the 14-3-3γ protein and consistent with the expression pattern of *TPD52L1* during the mucosal adenoma-carcinoma sequence. The 14-3-3γ protein is involved in many pathways, including the cell cycle, PI3K, and MAPK pathways (66, 67). hD53 expression is regulated by the cell cycle in breast cancer (13). hD53 was found to bind to 14-3-3 proteins by experimental phosphorylation of the 14-3-3 binding motif (41). 14-3-3 proteins play important roles in the cell cycle (68) and targeting *YWHAH* (14-3-3 eta) to enhance mitotic cell death presents a potential therapeutic strategy to overcome radioresistance in glioblastoma (69). It is possible that *TPD52L1* promotes tumor development by interaction with the 14-3-3γ protein.

Overall, the present RNAseq data provide important information to complement patient-matched transcriptomic profiles of the adenoma-carcinoma sequence. The detailed and comprehensive analysis provides information on the genetic features of the adenoma-carcinoma sequence, including canonical pathways, metabolism, and immune activities. Six specific dynamic expression patterns of DEGs associated with the time course of tumor formation were identified. Functional analysis of genes with different expression patterns revealed that suppression of the immune system occurred early during normal to adenoma transition, whereas activation of canonical pathways associated with cancer occurred during adenoma to carcinoma transition, and metabolic dysregulation was consistent throughout the transition process. Taken together with the results of WGCNA, these data indicate that genes with dynamic expression patterns were associated with tissue traits. DEGs in cluster 4 were highly associated with carcinoma traits and included many oncogenes, whereas DEGs in cluster 6 were highly associated with the traits of non-normal tissues, although these data were limited. RNAseq data combined with clinical information led to the selection of *TPD52L1* a prognostic factor for OS and DFS. Overexpression of *TPD52L1* promoted oncologic behaviors, including proliferation, colony formation, migration, and invasion. Consistently, *TPD52L1* downregulation inhibited tumor-associated behaviors. The proteomics results identified 70 proteins as the potential partners of *TPD52L1* in CRC. The pathways of the interacting proteins may help to clarify the mechanism of *TPD52L1* in tumor formation. These results suggest that the *TPD52L1* gene plays a key role in tumor formation.

## Data Availability Statement

The datasets generated in this study were upload to the Gene Expression Omnibus (GEO) under accession ID GSE164541. The datasets analyzed in this study are available in the GEO at https://www.ncbi.nlm.nih.gov/geo/query/acc.cgi?acc=GSE117606/.

## Ethics Statement

The studies involving human participants were reviewed and approved by the Ethics Committee of Zhongshan Hospital affiliated with the Shanghai Medical College of Fudan University. The patients/participants provided their written informed consent to participate in this study.

## Author Contributions

QH and BW conceived the project. QH designed experiments, analyzed the data, and drafted the manuscript. QH and ZL conducted the experiments. SC collected clinical samples. BL conducted the IHC analysis and collected clinical information. XC reviewed the manuscript. BW and YZ supervised the project. All authors contributed to the article and approved the submitted version.

## Funding

This work was supported by grants from the National Key R&D Program of China (grants 2018YFC1315000 and 2018YFC1315005) and the National Natural Science Foundation of China (grants 81672329 and 81861168036).

## Conflict of Interest

The authors declare that the research was conducted in the absence of any commercial or financial relationships that could be construed as a potential conflict of interest.

The handling editor declared a shared affiliation with the authors at time of review.

## Publisher’s Note

All claims expressed in this article are solely those of the authors and do not necessarily represent those of their affiliated organizations, or those of the publisher, the editors and the reviewers. Any product that may be evaluated in this article, or claim that may be made by its manufacturer, is not guaranteed or endorsed by the publisher.
