# Supplementary material for: Transcriptomic Analyses of the Adenoma-Carcinoma Sequence Identify Hallmarks Associated With the Onset of Colorectal Cancer
Source: Front Oncol. 2021 Aug 11;11:704531. doi: 10.3389/fonc.2021.704531 (PMC8387103; doi:10.3389/fonc.2021.704531)
Supplement: Supplementary file 1 [file DataSheet_1.docx]

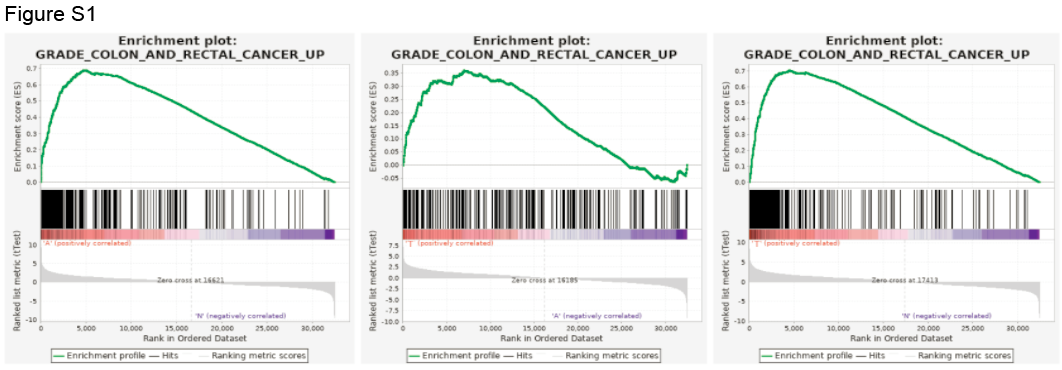


FigureS1: GSEA analysis on AN, MA and MN comparison, of with GRADE_COLON_AND_RECTAL_CANCER_UP dataset.


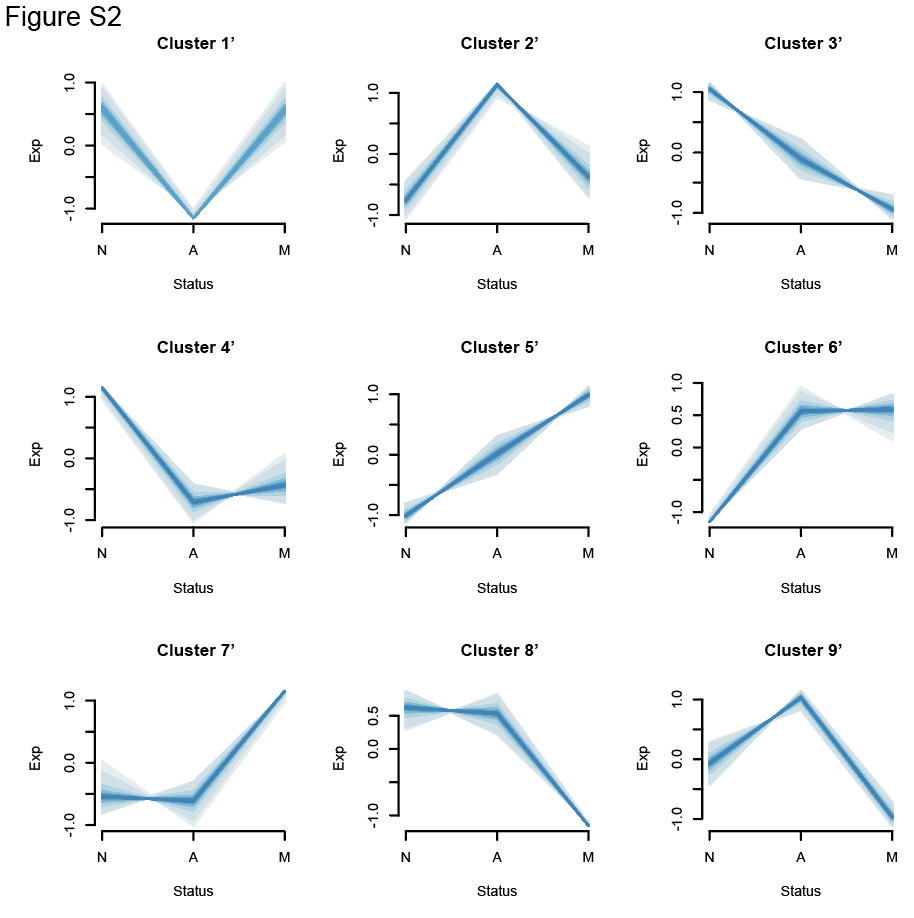
Figure S2: The expression patterns identified in GSE117606.


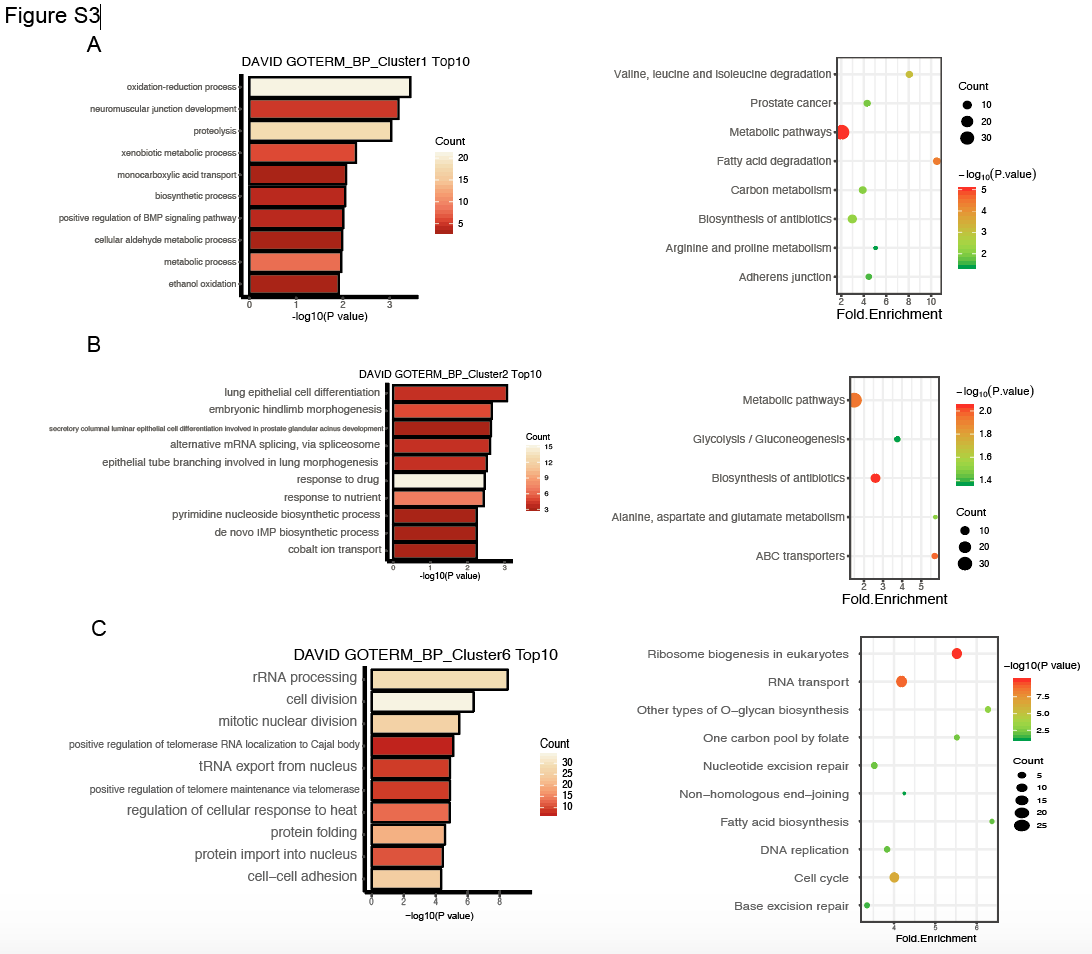


FigureS3: GO analysis of biological process and KEGG analysis on the genes of cluster 1 ,2 and 6.


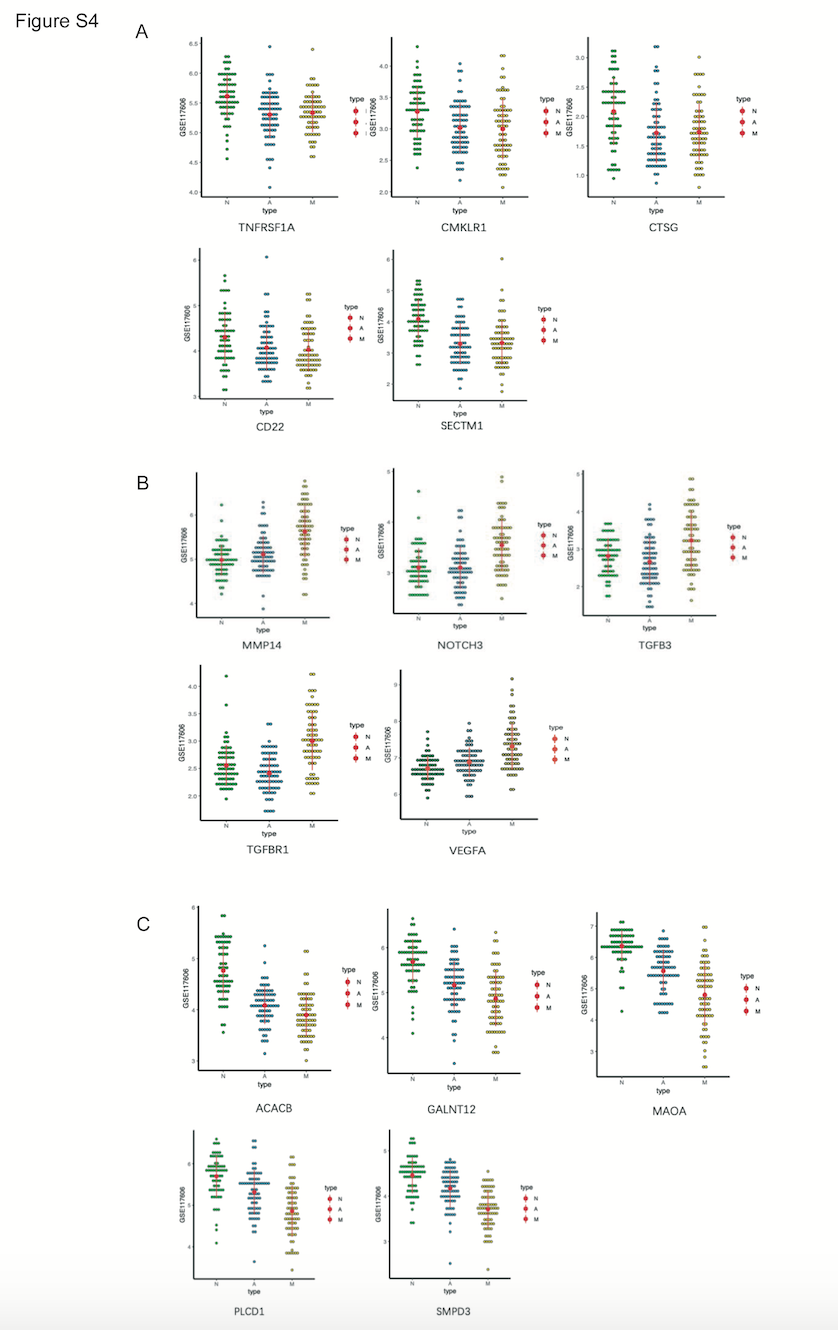


Figure S4: The biologic characteristics of the three clusters were validated by the GSE117606 dataset. (A) The genes associated with immune response in GSE117606 (TNFRSF1A, CMKLR1, CTSG, CD22, SECTM1) had similar expression patterns in cluster 3. (B) The genes associated with canonical cancer pathway in GSE117606 (MMP14, NOTCH3, TGFB3, TGFBR1, VEGFA) share the expression pattern with cluster 4. (C) The genes associated with metabolism in GSE117606 (ACACB, GALNT12, MAOA, PLCD1, SMPD3) had similar expression patterns in cluster 5.


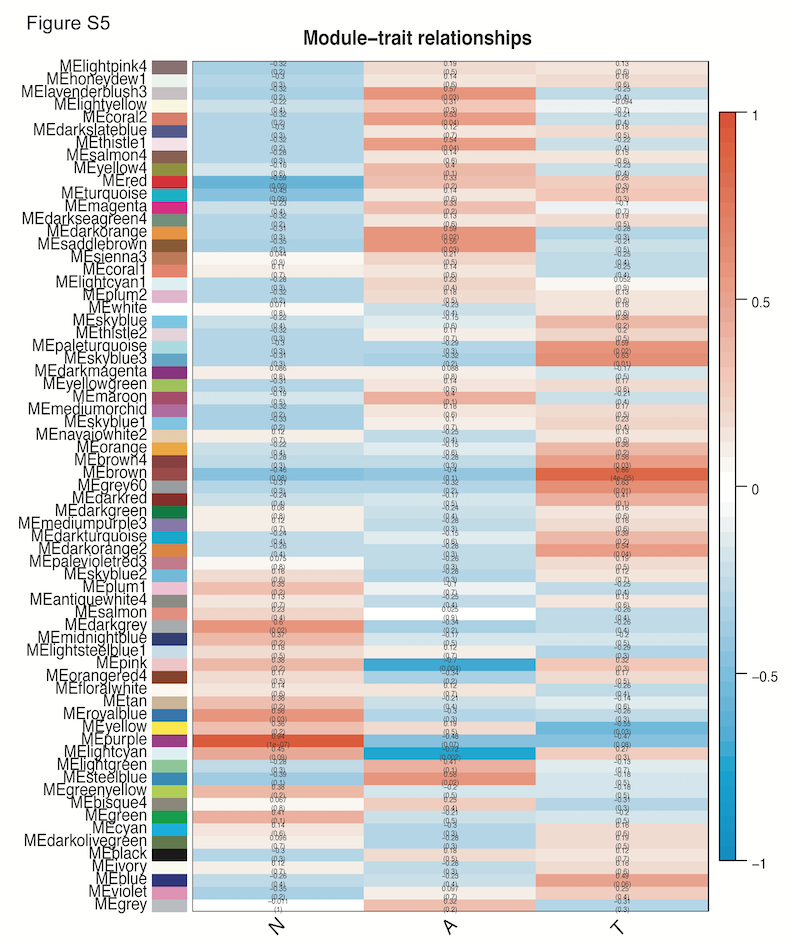


Figure S5: heatmap of revealed correlation ship and its p value between module and trait by WGCNA.


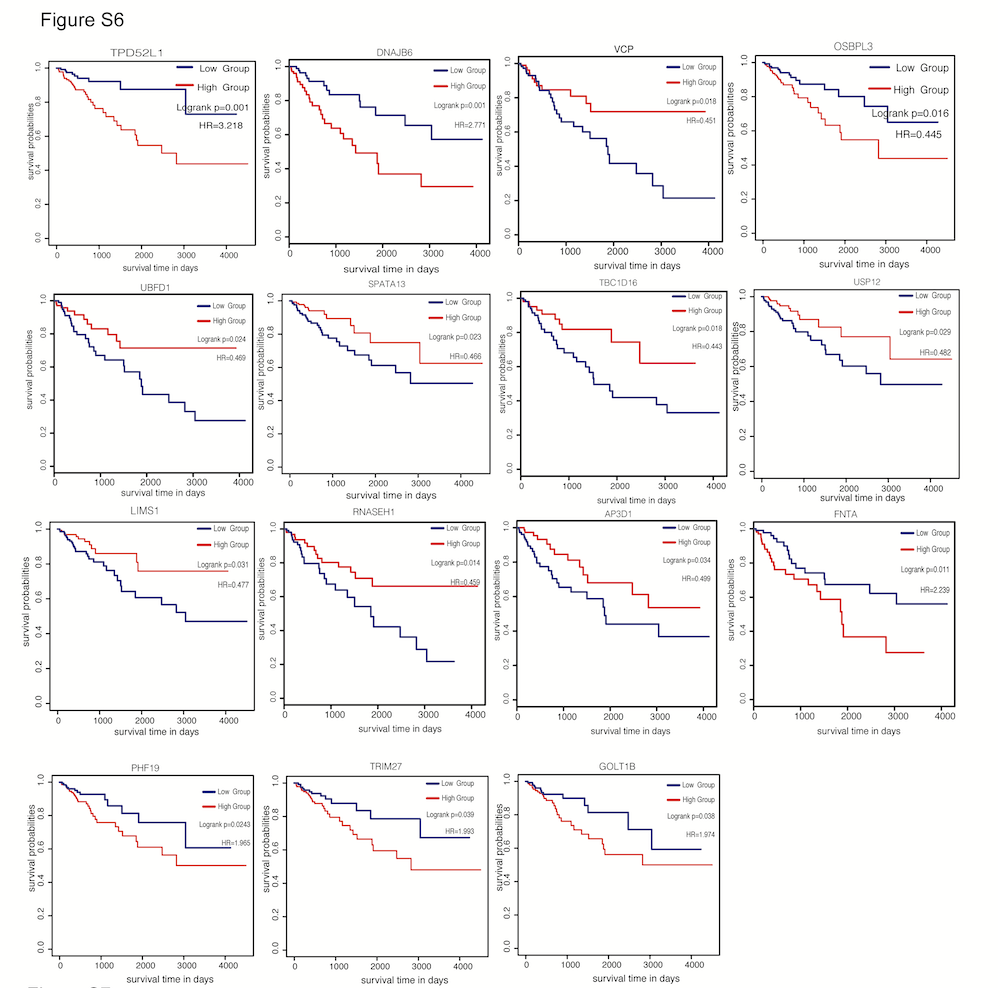


Figure S6: Kaplan-Meier survival curves of overall survival analysis on 15 genes with p<0.05 in cluster 6.


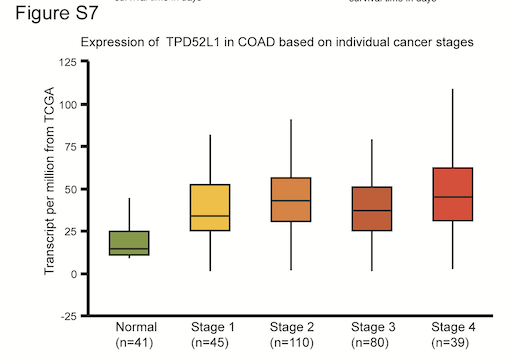


Figure S7: Boxplot of transcript level of TPD52L1 in different stages of COAD base on TCGA database.


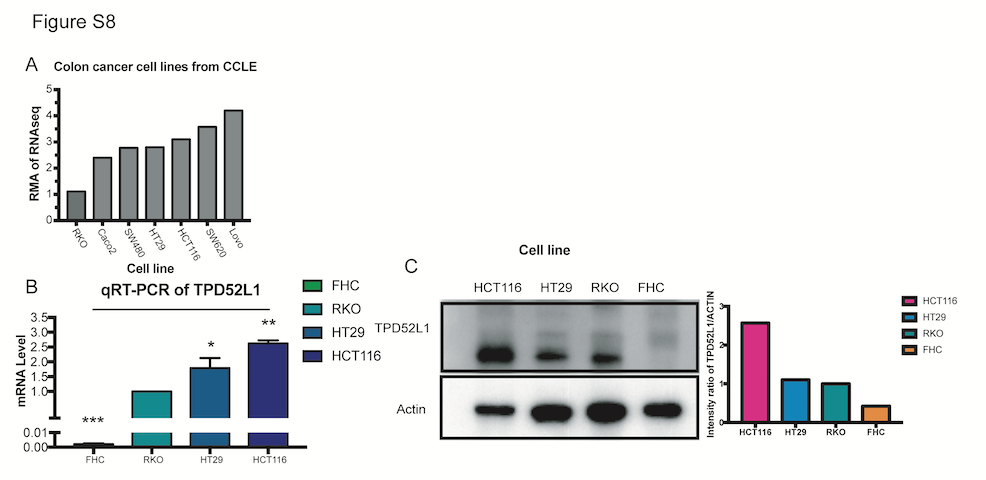


Figure S8: Expression level of TPD52L1 in different cell lines (A) RMA of TPD52L1 in 7 colorectal cancer cell lines from CCLE. (B)mRNA level of TPD52L1 were checked in FHC, RKO, HT29 and HCT116. (C) Protein level of TPD52L1 were checked in FHC, RKO, HT29 and HCT116.


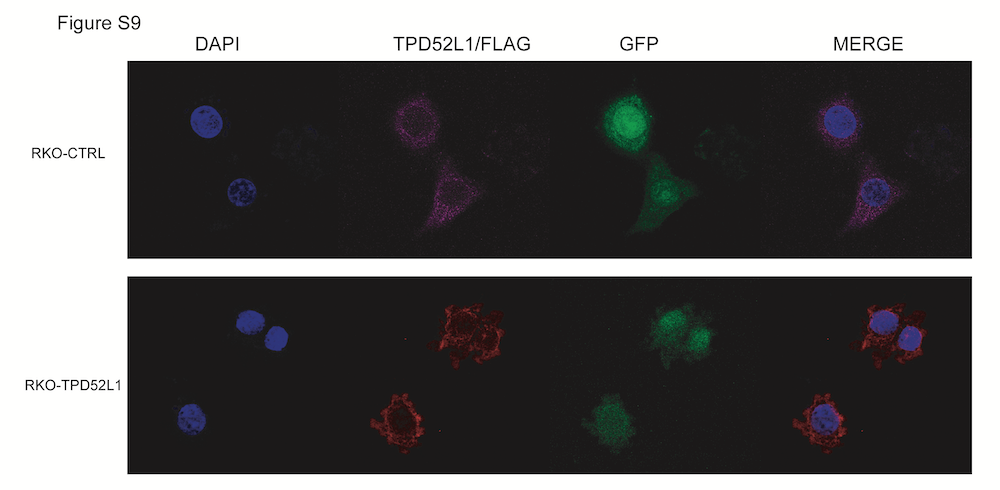


Figure S9: IF revealed the location of exogenous TPD52L1 is consistent with endogenous TPD52L1.


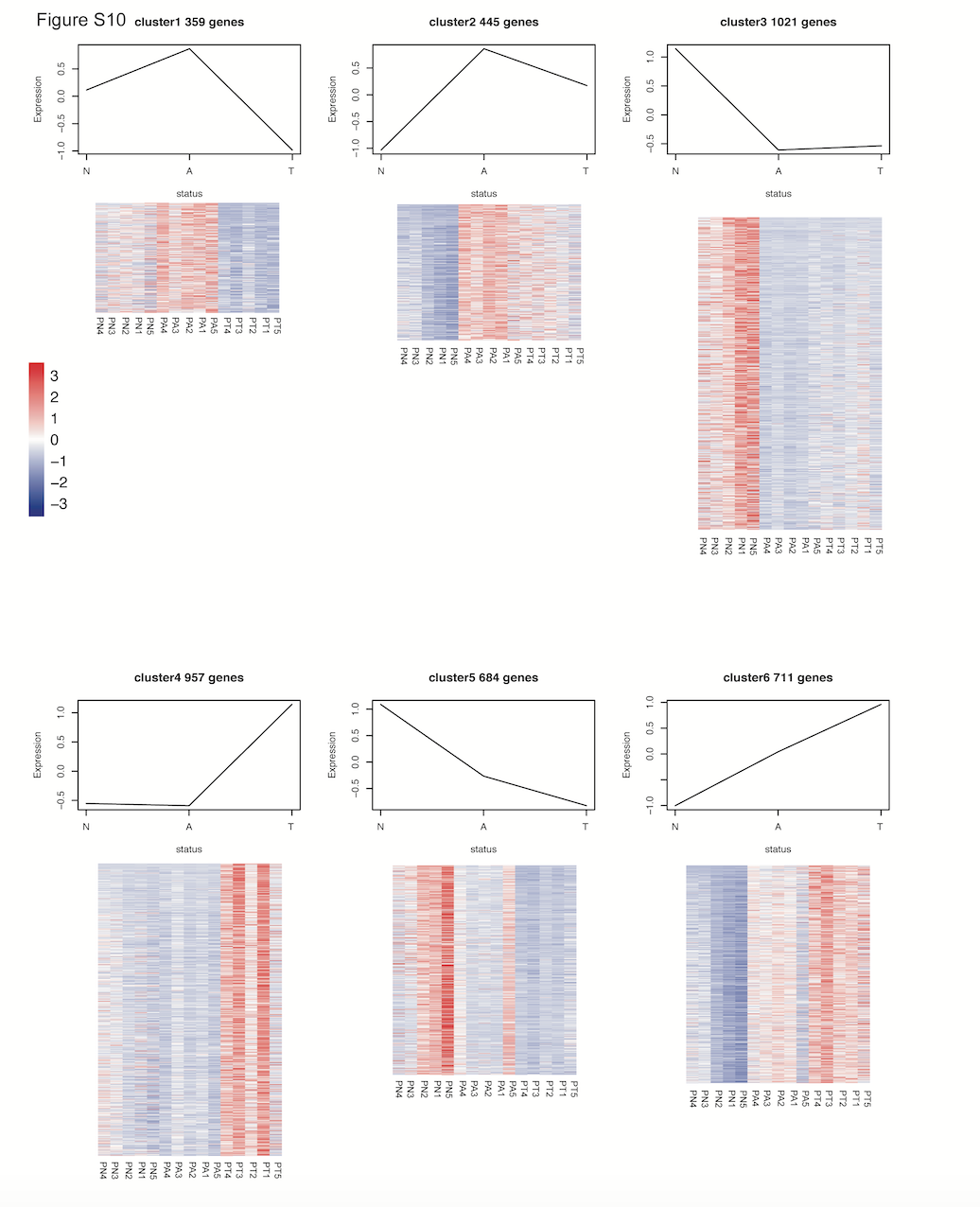


Figure S10: Heatmaps of expression levels of genes from six dynamic expression patterns in individual sample.


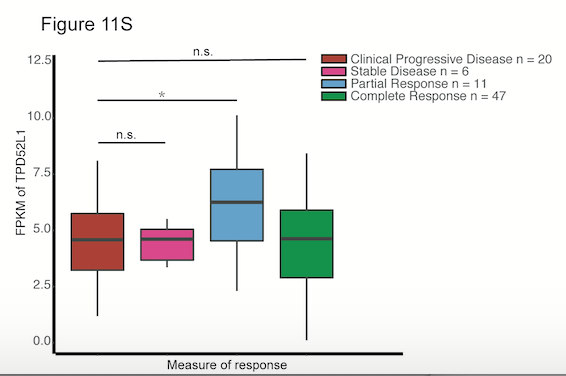


Figure S11: FPKM levels of TPD52L1 in the primary tumors with treatment response record.

Table S1: Clinical and histopathologic information for patient-matched normal colon, adenomas, primary tumor samples

Table S2: General RNA sequencing information of normal colon, adenomas, primary tumor samples

Table S3: DEGs of comparisons of adenoma vs. normal colon tissues (AN), primary cancer vs. adenoma tissues (MA), and normal colon vs. primary tumor tissues (MN)

Table S4: DEGs of comparisons of all three tissue types

Table S5: GSEA analysis of comparisons between any two of the three tissue types.

Table S6: DEGs in six dynamic expression patterns.

Table S7: 70 proteins were identified by the analysis of M.S. results of IP

Table S8: Total 110 pathways associated with 70 interacted proteins
